# Evaluation of the delta-shaped anastomosis in laparoscopic distal gastrectomy: midterm results of a comparison with Roux-en-Y anastomosis

**DOI:** 10.1007/s00464-014-3445-6

**Published:** 2014-02-12

**Authors:** Hidehiko Kitagami, Mamoru Morimoto, Masashi Nozawa, Kenichi Nakamura, Shinya Tanimura, Katsuhiko Murakawa, Yoshihiro Murakami, Kenji Kikuchi, Hajime Ushigome, Leo Sato, Minoru Yamamoto, Yasunobu Shimizu, Tetsushi Hayakawa, Moritsugu Tanaka, Satoshi Hirano

**Affiliations:** 1 Division of Endoscopic Surgery, Kariya Toyota General Hospital, 5-15 Sumiyoshi-cho, Kariya, Aichi 448-8505 Japan; 2 Department of Gastroenterological Surgery II, Hokkaido University Graduate School of Medicine, Sapporo, Hokkaido Japan

**Keywords:** Delta-shaped anastomosis, Laparoscopic distal gastrectomy, Intracorporeal gastroduodenostomy, Billroth I reconstruction

## Abstract

**Background:**

Various methods of reconstruction after laparoscopic distal gastrectomy (LDG) have been developed and published, whereas only a limited number of reports are available on the utility of the delta-shaped anastomosis (Delta). This study compared Delta and Roux-en-Y anastomoses (RY), with the aim to clarify the utility of Delta.

**Methods:**

Stage 1 gastric cancer patients who had undergone LDG with Delta (group D, *n* = 68) and those who had undergone LDG with RY (group RY, *n* = 60) were compared in terms of operative outcomes, postoperative clinical symptoms, gastrointestinal fiberscopic findings, and changes in body weight.

**Results:**

Both the operative and anastomotic times were significantly shorter in group D (230 and 13 min, respectively) than in group RY (258 and 38 min, respectively) (*p* < 0.001). Among the complications observed at the anastomotic site, obstruction was seen in one group D patient and two group RY patients but was relieved with conservative management. Postoperative clinical symptoms were reported for 26.4 % of the group D patients but had decreased to 5.9 % 1 year later. Group RY yielded similar results. Upper gastrointestinal fiberscopy performed 1 year postoperatively showed no intergroup differences in the incidence of gastritis or residual retention and a significantly more frequent occurrence of bile reflux in group D. Postoperative weight changes did not differ between the two groups.

**Conclusions:**

Delta reconstruction after LDG is a safe and effective procedure that is totally laparoscopic, less time consuming, and associated with a favorable postoperative course and a better quality of life.

Laparoscopic distal gastrectomy (LDG) for gastric cancer is known for its minimal invasiveness and better cosmesis. Hence, it is considered useful and continues to be an increasingly common option for gastric cancer treatment [1–5]. However, LDG techniques are yet to be standardized, including the number and location of ports, the lymph node dissection method, the use or omission of minilaparotomy, and the reconstruction method. Of these, the reconstruction method is the most likely to affect the postoperative quality of life (QOL) for the patient. Various techniques have been developed, and their outcomes reported [6–15].

The anastomotic procedures commonly used for reconstruction after LDG, as in cases of conventional laparotomy, are Roux-en-Y (RY), Billroth I (B–I), and Billroth II, with B–I and RY preferred. Several studies have compared B–I and RY to date, with reported findings showing the superiority of RY in terms of postoperative course [10, 11, 22]. However, opinions remain divided. Special techniques and approaches are needed to perform the aforementioned reconstructions under the LDG-specific visual field and under limited operability.

Numerous reports have been published on reconstruction using hand-sewn or stapled anastomoses with an adequate visual field ensured under laparoscopy or minilaparotomy. Basically, these techniques were originally used for open surgery, and each technique has its strengths and weaknesses. The surgeon’s preference determines which method and technique for reconstruction is used after LDG.

The delta-shaped anastomosis (Delta) was first reported by Kanaya et al. [6] in 2002 as an intracorporeal B–I anastomosis for LDG. It is an application of a functional end-to-end anastomosis used in operations of the small and large intestine to the anastomosis between the duodenal cut end and the gastric remnant, which is a unique technique not used in either LDG or open surgery.

Reports by Kanaya et al. [6, 7] indicate that Delta is an excellent reconstruction method performed under total laparoscopy that can be completed in a short time. However, the utility of Delta (as B–I) compared with RY has yet to be demonstrated. Given that Delta was developed to perform B–I in LDG, obtaining the advantages of B-1 over RY is a possibility that has not been obtained to date. It is necessary to validate whether adapting the delta anastomosis in LDG achieves the superiority of a less invasive procedure than RY for the patient including less blood loss, a shorter operation time, and a better postoperative QOL, which are noteworthy results that have been reported.

We have performed Delta reconstruction for LDG since 2003. This is the first comparative study of Delta and RY that reviews operative outcomes and postoperative courses of LDG after Delta and RY with the aim of clarifying the hypothesis that Delta is a safe and useful anastomosis.

## Methods

### Patients

Of 253 patients who underwent LDG between January 2008 and March 2011 at Kitami Red Cross Hospital or Kariya Toyota General Hospital, 165 patients with pathologic stage 1 gastric cancer who had been followed up for 1 year or more postoperatively by the end of April 2012 were selected for this study. The patients with Delta after gastrectomy performed by a single surgeon (H.K.) who specializes in this reconstruction method were assigned to group D (*n* = 68), whereas those with RY undertaken by another single surgeon (S. T.) who specializes in the RY reconstruction were allocated to group RY (*n* = 60). The two surgeons are both board-certified specialists of the Japan Society for Endoscopic Surgery. Their combined experience totaled more than 100 LDG cases before the aforementioned designated study subject selection period.

The status of the patients in both groups was classified according to the Japanese Classification of Gastric Carcinoma Version 13 (translation: 2nd English version) [16], and treatment was provided as per the Gastric Cancer Treatment Guidelines for Doctors’ Ref. [17].

### Operation methods

The operation was conducted with the patient in a leg-split position under general anesthesia. A 12-mm subumbilical video port was used. As operator ports, a 5-mm port in the right hypochondrium and a 12-mm port caudomedial to the first port were placed a fist width apart. As assistant’s ports, a 12-mm port in the left hypochondrium and another 12-mm port at the umbilical level caudomedial to the first port were placed.

**Fig. 1:**
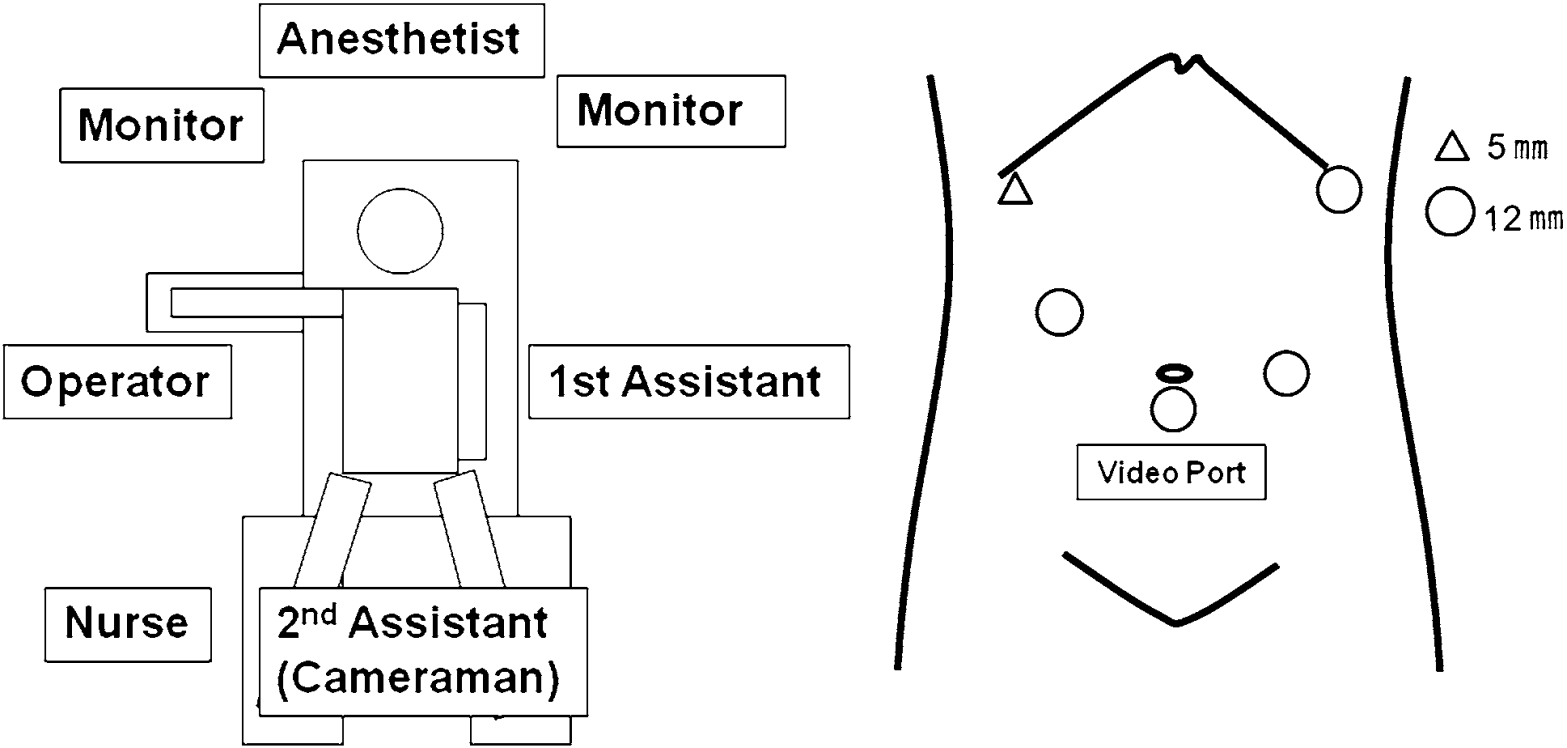
Patient position, arrangement in the operating room, and port sites

The operation was conducted at a carbon dioxide (CO_2_) insufflation pressure of 8 mmHg (Fig. 1). Either D1 + β or D2 lymph node dissection was performed [16, 17]. In all cases, the hepatic branch of the vagus nerve was conserved, whereas the celiac branch was resected. Gastrectomy with gastroduodenostomy or gastrojejunostomy was performed using 45- and 60-mm endoscopic linear staplers.

**Fig. 2:**
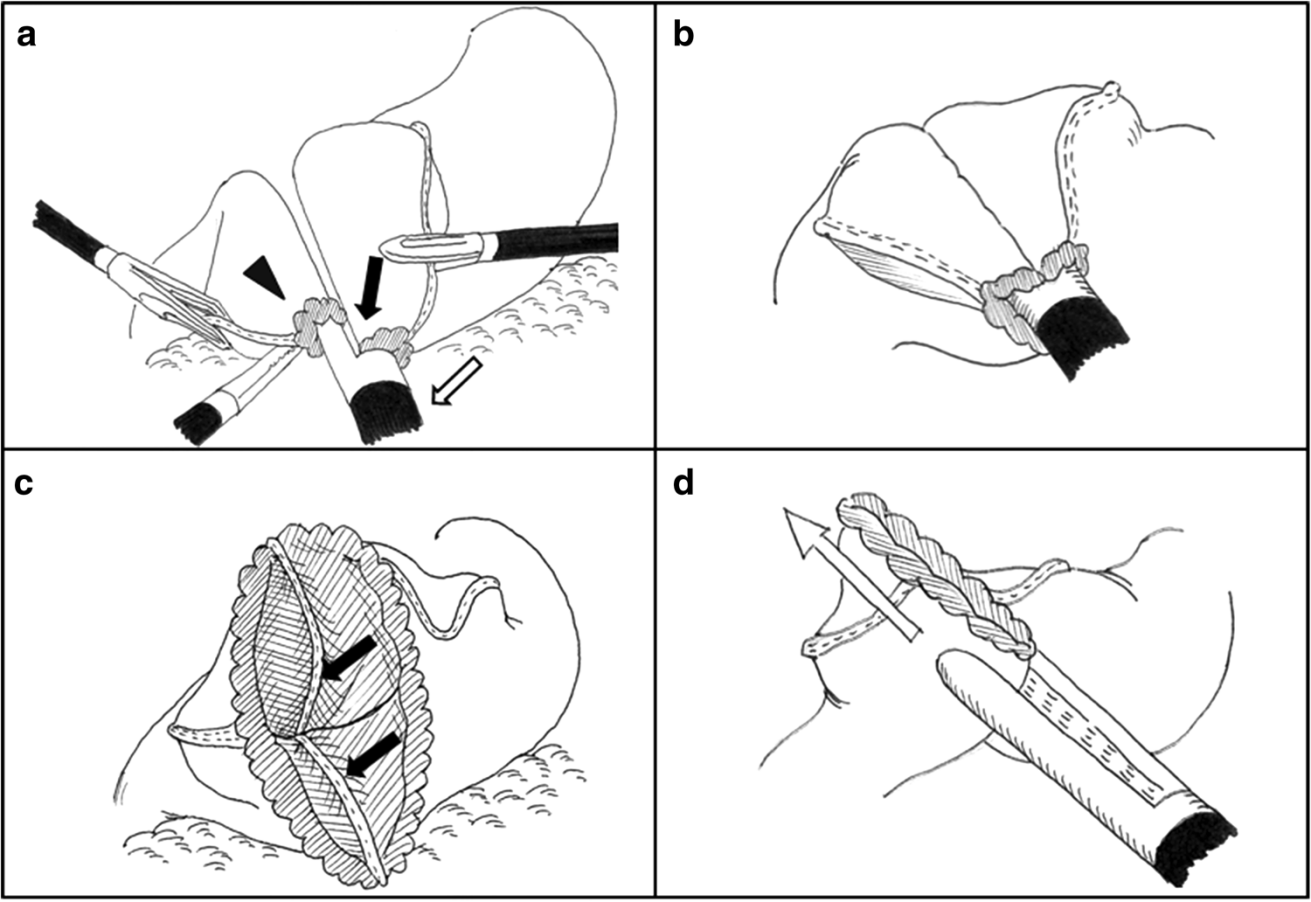
Schematic outline of the delta-shaped gastroduodenostomy. **A** Small incisions are created along the edge of the remnant stomach (*arrow*) and the duodenum (*arrowhead*), and the forks of the 45-mm endoscopic liner stapler (*white arrow*) are inserted. **B** The posterior walls of both the remnant stomach and the duodenum are approximated, and the stapler is fired. **C** The created V-shaped anastomosis (*arrows*). **D** The entry hole is closed by firing two more 45-mm staplers

Delta was performed according to the original procedure developed and reported by Kanaya et al. [6, 7]. The umbilical port wound was extended to about a 3-cm vertical incision, through which the resected stomach segment placed in a retrieval bag was extracted. The wound then was sutured back to the original port wound size and reinsufflated with CO_2_. A functional end-to-end anastomosis of the remnant stomach and the duodenal stump was created by firing a 45-mm linear stapler three times intracorporeally (Fig. 2). No additional postanastomotic sutures were used to strengthen the anastomotic site.

The following procedure was used for RY reconstruction. A 5-cm-long minilaparotomy was performed in the left upper abdomen or the epigastric region. The distal part of the stomach was exteriorized through the laparotomy, and a distal gastrectomy was undertaken extracorporeally under direct vision. The jejunum approximately 15 cm distal to the ligament of Treitz was extracted through the laparotomy and transected. A side-to-side jejunojejunostomy was constructed between the proximal jejunal stump and the distal jejunal portion about 20 cm below its cut end by firing a 45-mm linear stapler. The laparotomic opening was reinsufflated, and the distal jejunal stump was brought up to the gastric remnant in an antecolic manner. Gastrojejunostomy was performed by creating a side-to-side isoperistaltic anastomosis of the distal jejunal stump to the greater curvature of the remnant stomach using a 60-mm linear stapler. The entry hole was closed under direct vision through the laparotomy. In each patient, a closed drain was placed on the dorsal side of the remnant stomach as an information drain.

### Postoperative follow-up evaluation

All the patients were managed postoperatively in a similar manner following the critical path. The gastric tube was removed the day after surgery (on postoperative day [POD] 1), and ambulation training was initiated. Water intake was allowed, starting on POD 1.

On POD 3, contrast gastroradiography was performed for all the patients to examine the anastomotic site for leakage and food passage status. A liquid diet was started after the absence of any abnormalities had been confirmed. On POD 5, the drain was removed, and the patients were started on a soft diet. They were discharged between PODs 8 and 10. Thereafter, the patients were regularly followed up on an outpatient basis.

The patient characteristics of each group and the intra- and postoperative data of individual patients were assessed including operative time, anastomotic time, intraoperative blood loss, length of hospital stay, and complications. The anastomotic time, defined as the period from the initiation of the Delta procedure to the completion of anastomosis for group D and as the total time needed for jejunojejunostomy and gastrojejunostomy for Group RY, was computed based on the video feeds from the laparoscope, and the operative field camera was used intraoperatively.

The patients visited the outpatient department 1, 3, 6, 9, and 12 months after discharge and underwent body weight measurement and questioning regarding clinical symptoms. Patient medical records were thus created from the collected data and used to compare weight changes, symptoms (e.g., heartburn, heavy stomach feeling, reflux, dumping syndrome), and medication status between the groups.

Upper gastrointestinal fiberscopy (GIF) was performed 1 year after the operation, and the findings obtained were assessed in accordance with RGB (food *R*esidue, *G*astritis, *B*ile reflux) classification [18]. All data were expressed as median values. Statistical intergroup comparisons were undertaken with a Mann–Whitney U test or a *χ* ^2^ test using Fisher’s exact probability. All *p* values lower than 0.05 were considered significant.

## Results

Table 1 summarizes the patient characteristics of both groups (*n* = 128). The groups were well matched in terms of sex, mean age, and body mass index (BMI). The pathology of the excised specimens showed no intergroup differences in the percentage of stage 1A and 1B patients or in maximum tumor diameter. The length of the proximal margin (PM) and the distal margin (DM) from the tumor in the resected specimen [16] measured 52.2 and 70.9 mm respectively for group D while measuring 39.1 and 47.7 mm for group RY, with both the PM and DM lengths significantly longer (*p* < 0.001) in group D.

**Table 1:** Patient characteristics

	Group D^a^ (*n* = 68)	Group RY^b^ (*n* = 60)	*p* Value
Male/female	39/29	45/15	0.06
Age: years (range)	68.5 (45–89)	68.6 (44–87)	0.94
BMI (kg/m^2^)	23.7	22.5	0.07
Pathologic findings: stage IA/IB	56/12	44/16	0.22
Tumor size: mm (range)	30.0 (8–140)	24.6 (3–90)	0.06
PM/DM (mm)	52.2/70.9	39.1/47.7	<0.001

*BMI* body mass index, *PM* proximal margin, *DM* distal margin

Data are expressed as medians

^a^Group D underwent laparoscopic distal gastrectomy (LDG) with Delta anastomosis

^b^Group RY underwent LDG with Roux-en-Y anastomosis

Group D had a shorter median operative time (230 vs. 258 min) and a significantly lower median blood loss (21.5 vs. 50 mL) (*p* < 0.001) than group RY. The two groups did not differ in terms of lymph node dissection status (D1 + β and D2): group D (*n* = 57 and *n* = 11, respectively) and group RY (*n* = 44 and *n* = 16, respectively). The median anastomotic time for group D (*n* = 68) was 13 min, which was significantly shorter than for group RY (38 min; *n* = 42) (*p* < 0.001) (no video feeds from the operative field camera were available for 18 patients).

With regard to postoperative complications, one group RY patient experienced mesenteric hemorrhage, which necessitated a second operation, whereas no patients in group D required a repeat operation. Obstruction was observed in one group D patient and two group RY patients, all of whom were rehospitalized and placed under conservative management. The median hospital stay did not differ significantly between the two groups (9 days for group D vs. 10 days for group RY; Table 2).

**Table 2:** Operative data

	Group D^a^ (*n* = 68)	Group RY^b^ (*n* = 60)	*p* Value
Operative time (min)	230	258	<0.001
LN dissection: D1 + β/D2^c^	57/11	44/16	0.15
Anastomotic time (min)	13	38^d^	<0.001
Blood loss (mL)	21.5	50.0	0.003
Complications	Obstruction: 1	Obstruction: 2	
	Pancreas-associated: 1	Mesenteric hemorrhage: 1	
		Pancreas-associated: 2	
Hospital stay (days)	9	10	0.07

*LN* lymph node

Data are expressed as medians

^a^Group D underwent laparoscopic distal gastrectomy (LDG) with Delta anastomosis

^b^Group RY underwent LDG with Roux-en-Y anastomosis

^c^Classified according to Japanese classification of gastric carcinoma [16]

^d^ *n* = 42

The number of patients who presented to the outpatient department with heartburn, heavy stomach feeling, reflux, or dumping syndrome symptoms was 18 (26.4 %) in group D and 16 (26.7 %) in group RY, with no significant intergroup differences. Mosapride citrate hydrate, proton pump inhibitors, or H2 blockers were prescribed as appropriate for symptom relief. Symptoms persisted for 1 year for four group D patients (5.9 %) and six group RY patients (10 %), with only four patients in group RY (6.7 %) requiring medication. Dumping syndrome symptoms were reported for two group D patients (2.9 %) and one group RY patient (1.6 %) but improved solely with dietary instruction (Table 3).

**Table 3:** Postoperative symptoms

	Group D^a^ (*n* = 68)	Group RY^b^ (*n* = 60)	*p* Value
Symptoms	18 (26.4)	16 (26.7)	0.98
Heartburn	8	3	
Heavy stomach feeling	11	12	
Reflux	12	12	
Dumping syndrome	2	1	
Continuation of symptoms 1 year later: *n* (%)	4 (5.9)	6 (10)	0.59
Continuation of medication to treat complaints: *n* (%)	0	4 (6.7)	0.1
		Mosapride citrate hydrate: 3	
		PPI: 1	
		H2 blocker: 1	

*PPI* proton pump inhibitor

Values express numbers of cases; symptoms include overlap

^a^Group D underwent laparoscopic distal gastrectomy (LDG) with Delta anastomosis

^b^Group RY underwent LDG with Roux-en-Y anastomosis

**Fig. 3:**
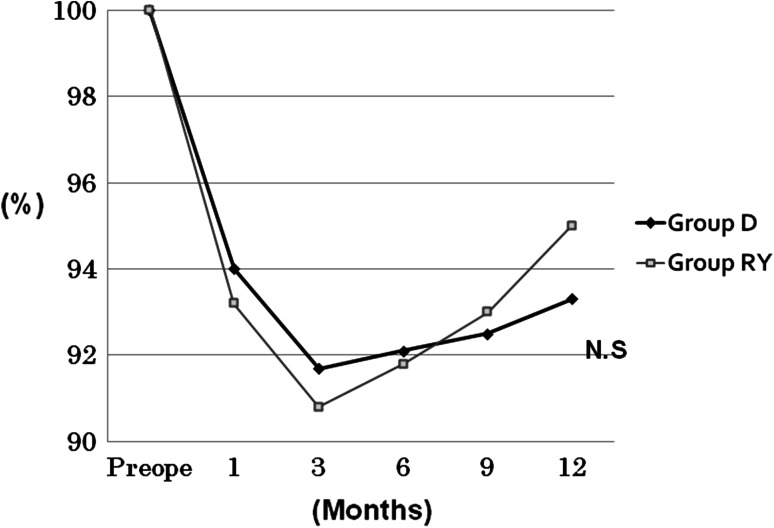
Weight changes over the first postoperative year. No significant intergroup difference was observed at any time point. *Horizontal axis*: time from the operation. *Preope* preoperative. *Vertical axis*: median % weight at 1, 3, 6, 9, and 12 months, with the preoperative weight as 100 %

Weight change did not differ between the two groups at any of the designated postoperative time points of 1, 3, 6, 9, and 12 months. Both groups exhibited a tendency toward weight gain at 6 months and thereafter (Fig. 3).

**Fig. 4:**
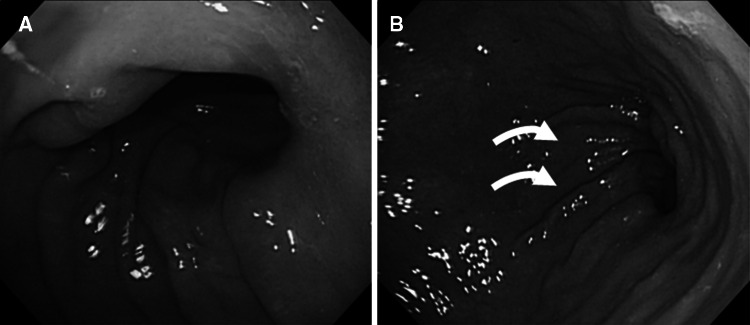
Upper gastrointestinal fiberscopy 1 year after Delta. **A** Closeup image of the anastomosis. The opening is an oval with a sufficiently large diameter. **B** Distant image of the anastomosis. Neither gastritis nor food residue is observed. The opening is twisted dorsally (*arrows*)

Upper gastrointestinal fiberscopy was performed 1 year postoperatively for 51 patients (75 %) in group D and for 48 patients (80 %) in group RY. The GIF findings for group D included a large-diameter, oval-shaped anastomotic opening, which was twisted dorsally (Fig. 4). The findings were assessed as either grade 0 or 1 or higher according to the RGB classification. No intergroup differences in terms of food residue or gastritis were detected. The number of patients with bile reflux was significantly higher in group D (*n* = 30, 58.8 %; *p* < 0.001; Table 4).

**Table 4:** RGB scores

	Group D^a^ (*n* = 51) *n* (%)	Group RY^b^ (*n* = 48) *n* (%)	*p* Value
Food residue			
Grade 0	37 (72.5)	28 (58.2)	0.14^c^
1	10	11	
2	3	8	
3	1	1	
4	0	0	
Gastritis			
Grade 0	21 (41.2)	28 (59.4)	0.09^c^
1	25	18	
2	5	2	
3	0	0	
4	0	0	
Bile reflux			
Grade 0	21 (41.2)	38 (79.2)	<0.001
1	30	10	

*RGB* food residue, gastritis, bile reflux [18]

^a^Group D underwent laparoscopic distal gastrectomy (LDG) with Delta anastomosis

^b^Group RY underwent LDG with Roux-en-Y anastomosis

^c^Score 0 versus 1 and higher

## Discussion

Laparoscopic distal gastrectomy is rapidly becoming a common option for gastric cancer treatment. Although a variety of techniques have been developed for LDG [1–15], a standard reconstruction method and techniques for conducting a safe operation are yet to be established, making their standardization essential for more widespread use of this procedure.

The RY and B–I approaches are commonly used for reconstruction after LDG. The RY procedure is reported to be associated with a lower incidence of anastomotic leakage and a favorable postoperative QOL [10, 11]. However, this procedure is laborious, requiring two anastomoses and one duodenal stump closure, and involves a risk of internal hernia and Roux stasis syndrome, among other complications.

The B–I procedure, on the other hand, is advantageous in that it requires only a single anastomosis, retains physiologic food passage, and poses no risk of internal hernia. However, the disadvantages of B–I reconstruction after LDG include a higher risk of anastomotic leakage compared with RY and difficulty securing a satisfactory visual field under a minilaparotomy due to limited mobility of the duodenal stump [2, 4, 5, 19, 20].

In contrast, Delta, a totally laparoscopic intracorporeal procedure completed with a linear stapler alone, offers the advantages of being virtually unaffected by patients’ physical constitution in securing of the visual field and less subject to variation in the surgeon’s suturing skill level. Kanaya, the developer of Delta, together with colleagues [7] reviewed the outcomes of their first 100 consecutive Delta cases and obtained the following findings. Surgeons were able to master the learning curve quickly and the required skills for the procedure, and patients were discharged early with adequate food passage after surgery, with only one case of complications (minor leakage) and minimal damage to the abdominal wall. They concluded that Delta was a useful anastomosis reconstruction technique based on the aforementioned observations and a good postoperative QOL, as indicated by adverse event reports consisting of only mild complaints and dumping syndrome (1.3 %) during the outpatient follow-up period.

Sharing the same opinion, we conducted Delta under the assumption that Delta can be performed in a short operative time under full laparoscopy. We believed that the postoperative course should bring better results as well and that the different results should be seen when RY and conventional B-1 are compared.

The current study showed that the median operative time for Delta was 13 min, which was significantly shorter than for RY. A reconstruction method that can be completed in 10-plus minutes after gastrectomy is appealing for health care providers. In addition, it differed favorably in both blood loss and operative time, attributable to the absence of both an abdominal incision and mesenteric handling, and to the minimal number of anastomosis required. From the patient’s perspective, Delta is a less invasive and thus more beneficial procedure with a shorter operative time and less blood loss.

Regarding anastomotic-site complications, obstruction was reported in one Delta patient and two RY patients. Anastomotic leakage, the most significant problem with anastomosis, did not occur in either group. The possible causes of anastomotic leakage and obstruction include tension and blood circulation at the anastomotic site.

Anastomotic-site tension, related to the size of the gastric remnant, is a particular problem with B–I. Although it is difficult to measure the size of the post-LDG remnant stomach, the specimens in the current study showed a longer PM and DM with Delta than with RY, indicating that the remnant stomach was smaller on the average in Delta patients.

We contend that with RY, a visual field secured under a minilaparotomy makes it possible for the surgeon to locate the tumor and to perform gastrectomy to just the extent necessary, whereas with Delta, difficulty identifying the tumor’s location under total laparoscopy results in the removal of two-thirds to three-fourths of the stomach, as is typical in a standard gastrectomy.

The aforementioned finding indicates that with Delta it is not necessary to try leaving as large a segment of the stomach as possible in consideration of postgastrectomy tension and that standard distal gastrectomy will suffice, with the anastomotic site capable of withstanding the resulting tension. Regarding circulation at the anastomotic site, Delta carries a risk of ischemia in the duodenal stump because the tissue around it is dissected to prepare a margin for suturing, and also because of the anastomotic alignment that results from the reconstruction.

Computed tomography performed during rehospitalization showed that the Delta patient who experienced obstruction had edema and panniculitis at the anastomotic site. Transient edematous narrowing at the anastomotic site appeared to be a probable cause of these events. Because edematous narrowing is likely associated with impaired circulation, excessive dissection of periduodenal tissue should be avoided in Delta to prevent ischemia.

Obstruction also occurred in two RY patients, but in contrast to B–I cases, stasis in the jejunal arm was suspected as a probable cause [21]. In early gastric cancer cases, the greater omentum outside the dissection area is preserved, and with Delta, unlike RY, the intestinal tracts inside the greater omentum can be left unmanipulated. Therefore, Delta is less associated with postoperative events such as bowel dysfunction including stasis, internal hernia, and mesenteric hemorrhage. We have been maintaining the jejunal arm for stasis prevention in RY as 20 cm short. The result is that the remnant stomach has been larger on the average in RY patients, with gastrectomy performed under the direct vision of minilaparotomy. In future studies, it also is necessary to examine whether there is a possible contributor to increased delayed gastric-emptying symptoms. Because of the aforementioned factors, Delta is an anastomosis method that can be quickly and safely conducted, as well as a procedure that withstands tension to the extent that caution is exercised against the possible development of duodenal ischemia.

Upper gastrointestinal fiberscopy showed that Delta is an anastomosis with a spacious opening. This may lead to problems such as postoperative gastritis secondary to reflux of bile and pancreatic juice from the duodenum and dumping syndrome due to rapid food passage.

In the current study, Delta and RY did not differ in terms of clinical symptoms or medication. Dumping syndrome symptoms were reported in only 2.9 % of the Delta patients and improved with dietary instruction. Upper gastrointestinal fiberscopy showed a significantly greater number of patients with bile reflux in the Delta group, whereas the incidence of postoperative gastritis and food residue did not differ significantly between the groups.

Kanaya et al. [7] also reported that the higher bile reflux incidence in Delta patients was unrelated to clinical symptoms. In addition, these authors noted transient retention of contrast medium at the anastomotic site from contrast gastroradiography performed several months after a Delta operation, as opposed to rapid passage observed soon after the operation. It was suspected that this transition in passage conditions is the reason for the low incidence of dumping syndrome symptoms in Delta patients.

Other researchers comparing RY and B–I have reported an association of B–I with more frequent occurrences of dumping syndrome symptoms as well as bile reflux and postoperative gastritis, as shown by GIF [10, 11, 22]. The B–I assessed in these studies involved circular-stapled or hand-sewn anastomosis, unlike the procedures with Delta. Delta may entail certain mechanisms to prevent rapid passage and reflux, which may explain the inconsistency between the aforementioned findings obtained with B–I and our results with Delta.

Upper gastrointestinal fiberscopy showed the presence of dorsal twisting at the Delta anastomotic site. In addition to the twisting between the remnant stomach and the duodenum caused at the time of reconnection in Delta, the stomach is suspected to expand and lean anteriorly after food intake, imposing a further twist to the anastomotic site and thereby preventing rapid food passage. During fasting, which is when GIF is undertaken, bile reflux may occur through the wide anastomotic opening, but after food ingestion, dumping syndrome is expected to be less likely due to greater twisting at the anastomotic site and the resulting food retention.

Regarding weight changes, a similar level of weight gain was observed in both groups. This likely was attributable to the absence of differences in postoperative food intake conditions due to an absence of differences in postoperative clinical symptoms. Based on the aforementioned findings, Delta does not differ from RY in the course of the first postoperative year and does not pose any significant problems for QOL.

Delta has been gradually gaining popularity in recent years, with improved techniques as well as short- and long-term favorable outcomes reported from several studies [7, 12, 20, 23, 24]. The results of the current study comparing Delta with RY demonstrated that Delta is a useful anastomotic procedure because the postoperative course and QOL, observed to be better in RY than in conventional B–I, was not inferior to RY. In addition, Delta had an evidently shorter operative time.

The current study, however, was a retrospective cohort study biased in several respects including its involvement of interoperator differences and its restriction to early cancers as the target disease. In addition, a limitation existed in that internationally validated questionnaires were not used for evaluation of postoperative clinical symptoms. Moreover, due to the limited number of cases, the observation period was for only 1 year. By extending the observation period and by collecting more cases, new results may be obtained. Further investigation of Delta in a randomized clinical trial setting is needed to validate its safety and feasibility.

## Disclosures

Hidehiko Kitagami, Mamoru Morimoto, Masashi Nozawa, Kenichi Nakamura, Shinya Tanimura, Katsuhiko Murakawa, Yoshihiro Murakami, Kenji Kikuchi, Hajime Ushigome, Leo Sato, Minoru Yamamoto, Yasunobu Shimizu, Tetsushi Hayakawa, Moritsugu Tanaka, Satoshi and Hirano have no conflicts of interest or financial ties to disclose.
